# Effects of the inclusion of physical activity in secondary education academic classes on educational indicators and health markers: rationale and methods of the ACTIVE CLASS study

**DOI:** 10.3389/fpubh.2023.1329245

**Published:** 2024-01-05

**Authors:** María González-Pérez, David Sánchez-Oliva, Alberto Grao-Cruces, Enrique Cano-Cañada, Fátima Martín-Acosta, Raúl Muñoz-González, Francisco J. Bandera-Campos, Abel Ruiz-Hermosa, Miguel Vaquero-Solís, Carmen Padilla-Moledo, Julio Conde-Caveda, Víctor Segura-Jiménez, Inmaculada González-Ponce, Tomás García-Calvo, José Castro-Piñero, Daniel Camiletti-Moirón

**Affiliations:** ^1^GALENO Research Group, Department of Physical Education, Faculty of Education Sciences, University of Cadiz, Puerto Real, Spain; ^2^Instituto de Investigación e Innovación Biomédica de Cádiz (INiBICA), Cadiz, Spain; ^3^ACAFYDE Research Group, Department of Didactics of Musical, Plastic and Body Expression, Faculty of Sports Sciences, University of Extremadura, Cáceres, Spain; ^4^Social and Health Care Research Center, University of Castilla-La Mancha, Cuenca, Spain; ^5^UGC Medicina Física y Rehabilitación, Hospital de Neurotraumatología y Rehabilitación, Hospital Universitario Virgen de las Nieves, Granada, Spain; ^6^Instituto de Investigación Biosanitaria ibs.GRANADA, Granada, Spain

**Keywords:** physically active learning, active breaks, adolescents, physical fitness, sedentary time, academic indicators, cognition, motivational variables

## Abstract

**Background:**

Increasing physical activity (PA) levels and reducing sedentary behaviors in children and adolescents is a need, especially in schools. Active breaks and physically active learning are examples of two emerging methodologies that have been shown to be effective in increasing PA levels and additionally produce improvements in children’s educational markers. However, the evidence in adolescents is very limited. This paper presents the design, measurements, and interventions implemented in the ACTIVE CLASS study, whose objectives are: (i) evaluate the effects of two interventions on PA levels, sedentary time, health-related physical fitness academic indicators, cognition, and markers of psychological health among secondary education students; (ii) evaluate teachers’ and students’ experiences about the implementation of these the two school-based PA intervention.

**Methods:**

A randomized controlled study is conducted with a total of 292 students aged 12–14 years old from six schools (7th and 8th grade) in Spain (three in Cadiz and three in Caceres). One school from each study provinces is randomly assigned to either the active break intervention group, the physically active learning intervention group, or the control group. The interventions have a duration of 16 weeks. Nine main measurement categories are assessed: PA and sedentary time, health-related physical fitness, academic indicators, cognition, psychological health, motivational variables, dietary patterns, sociodemographic characteristics, as well as qualitative information through semi-structured individual interviews and focus groups. Three independent measurements of evaluation are distinguished: pre-intervention, post-intervention (week 16) and retention measurement (4 weeks after the intervention). For quantitative variables, descriptive, correlational, regression and repeated measures ANOVA will be applied.

**Discussion:**

To the best of our knowledge, the ACTIVE CLASS study is the first of its kind in Spain to evaluate the effects of incorporating active breaks and physically active learning in secondary education. In addition, this project provides important information on the effects of two school-based PA intervention arms on educational variables and health markers in adolescents. This will provide valuable and innovative training to the educational community, enabling them to implement teaching methodologies that have the potential to enhance academic performance and improve the quality of life for their students.

**Clinical trial registration:**

clinicaltrials.gov, NCT05891054.

## 1 Introduction

Physical activity (PA) in children and adolescents has not only been consistently associated with physical health (i.e., adiposity, cardiometabolic health, health-related physical fitness (HRPF), bone health, etc.) (1), but its impact extends to psychosocial health (i.e., well-being, self-image, anxiety, depression, life satisfaction, happiness, etc.) (2). Consequently, the World Health Organization recommends that children and adolescents aged 5 to 17 should accumulate at least 60 min of moderate-to-vigorous PA (MVPA) per day (3). However, there is a concerning scenario in Europe, where the majority of adolescents, exceeding 71%, fail to meet these PA guidelines (4). This lack of adherence to PA guidelines has significant implications for the development of non-communicable diseases.

Schools serve as environments where millions of children and adolescents, representing diverse socioeconomic backgrounds, cultures, and fitness profiles, coexist for extended periods of time. This makes the school an ideal setting for developing strategies to encourage the adoption of healthy lifestyle habits. Moreover, the American Heart Association recommends that children and adolescents should accumulate at least 30 min of MVPA in the school day (5). Nevertheless, a recent systematic review revealed that less than a quarter of children and adolescents worldwide meet these recommendations for PA during school hours (6). On average, children and adolescents accumulate only 3 to 22% and 2 to 8% of their school time in MVPA, respectively (6). Moreover, sedentary behaviors are prevalent during the school day, with children and adolescents spending a significant amount of time sitting at their desks (7, 8). Thus, the Sedentary Behavior Research Network recommends replacing screen-based learning activities with screen-free activities, preferably incorporating movement (9).

Currently, schools serve as alarming hubs of inactivity and sedentary behavior, necessitating strategies that aim to increase MVPA levels and reduce prolonged sedentary periods during the school day. Traditionally, physical education lessons and recess have been regarded as opportune periods to incorporate PA during the school day. However, such methods have proven ineffective in increasing PA levels, particularly among adolescents (10). As a result, there has been a growing interest in recent years in developing strategies to enhance PA levels during subjects traditionally taught in a static manner within the classroom, such as mathematics, geography, and history. Two such strategies are active breaks (AB) and physically active learning (PAL) (11, 12).

AB involve incorporating short breaks of PA (usually with moderate or vigorous intensity) into academic lessons, excluding physical education (13). Previous research has predominantly focused on the effects of AB on PA levels and educational outcomes. Notably, AB ranging from 5 to 15 min have been associated with improved PA levels during school hours (14–17). Regarding educational variables, the impact of AB on time-on-task has been investigated, and it appears to be significantly higher when AB are integrated (14, 16, 17). The results on other variables such as academic and cognitive performance are inconclusive (16, 17), likely due to the variability in measurement tools used across studies. It is important to note that most of the evidence on AB comes from studies conducted in primary education, and there is limited research on the effectiveness of this type of intervention in secondary education. Few studies that have evaluated the effectiveness of AB in secondary education have reported conflicting results regarding to the impact on time-on-task (18, 19). In relation to cognitive variables, it seems that AB of 4 min duration for 8 weeks is sufficient to significantly improve attention, concentration and mathematical calculation (20).

PAL involves integrating PA into lessons in key learning areas other than physical education (e.g., mathematics) (13). Previous studies have suggested positive effects of PAL on PA levels, time-on-task and academic performance in primary education children (21). However, there is no clear evidence regarding to the changes on HRPF, with some studies reporting significant effects (22, 23) while others do not (21). Likewise, as in AB, the effects of this type of intervention on cognitive markers are inconclusive (21). Once again, the available evidence in secondary education remains limited and inconclusive. PAL seems to be linked with enhancements in school-based PA levels, but not in overall PA (24). Moreover, there are contradictory findings regarding the impact of PAL on academic performance (25, 26). Additionally, there is a need for studies in this population that include other variables known to have a positive association with PA, such as time-on-task (27) and cognitive variables (28).

The scarcity of studies and the disparity of results found in secondary education, joined with the observed decline in PA levels during this stage (29, 30), underscore the importance of implementing interventions to promote and increase PA in the classroom. Moreover, if we focus on the Spanish context, to date no such interventions based on AB or PAL have been carried out in the secondary education classroom.

Thus, this study reports on the methods and rationale of the ACTIVE CLASS study. This randomized controlled trial (RCT) has two main objectives: (i) examines the effect of two active learning intervention (AB and PAL) on PA levels, sedentary time, educational indicators, cognition, and markers of physical and psychological health in secondary education students; (ii) evaluate teachers’ and students’ experiences about the implementation of these the two school-based PA intervention.

## 2 Methods and analysis

### 2.1 Study design

The ACTIVE CLASS study is a RCT, with randomization at school level [Clinicaltrials.gov (NCT05891054)]. An intervention is developed in two experimental groups (AB intervention and PAL intervention), with pre- and post-intervention measures. Additionally, a follow-up measure is conducted 4 weeks after the intervention to assess the sustainability of the intervention program.

ACTIVE CLASS is a multicenter study whose management is designed to ensure effective collaboration and communication between research groups. The research groups consist of qualified researchers and graduates in PA and sport sciences from two Universities (University of Cadiz and University of Extremadura, Spain). Both Research groups adhere to a common study protocol for training, fieldwork, data collection and management, and quality control procedures. There is continuous telematic contact during the development of the study. In addition, each research group has responsible investigators who manage the day-to-day running of the study in their centers and are in continuous contact with their counterparts at the other university to make decisions on aspects that require a quick response or do not require a meeting of all the in researchers.

### 2.2 Participants and inclusion criteria

Participants in the ACTIVE CLASS study are apparently healthy adolescents from secondary schools in the provinces of Cadiz and Caceres (Spain). The schools included in the study are public secondary schools located in areas with a predominantly medium socioeconomic level (31). The ACTIVE CLASS study establishes the following inclusion criteria:

- *Participants’ inclusion criteria for adolescents’ students:* (i) studying 7th or 8th grade (12–14 years old); (ii) not having a physical disability or health problem that could limit PA levels.
- *Schools’ inclusion criteria:* (i) having a minimum of 60 students in 7th and 8th grade; (ii) not participating in any other PA or health promotion program; (iii) Be located within a radius of 15 kilometers of the research group’s work centers in Cadiz and Caceres.

**Figure 1:**
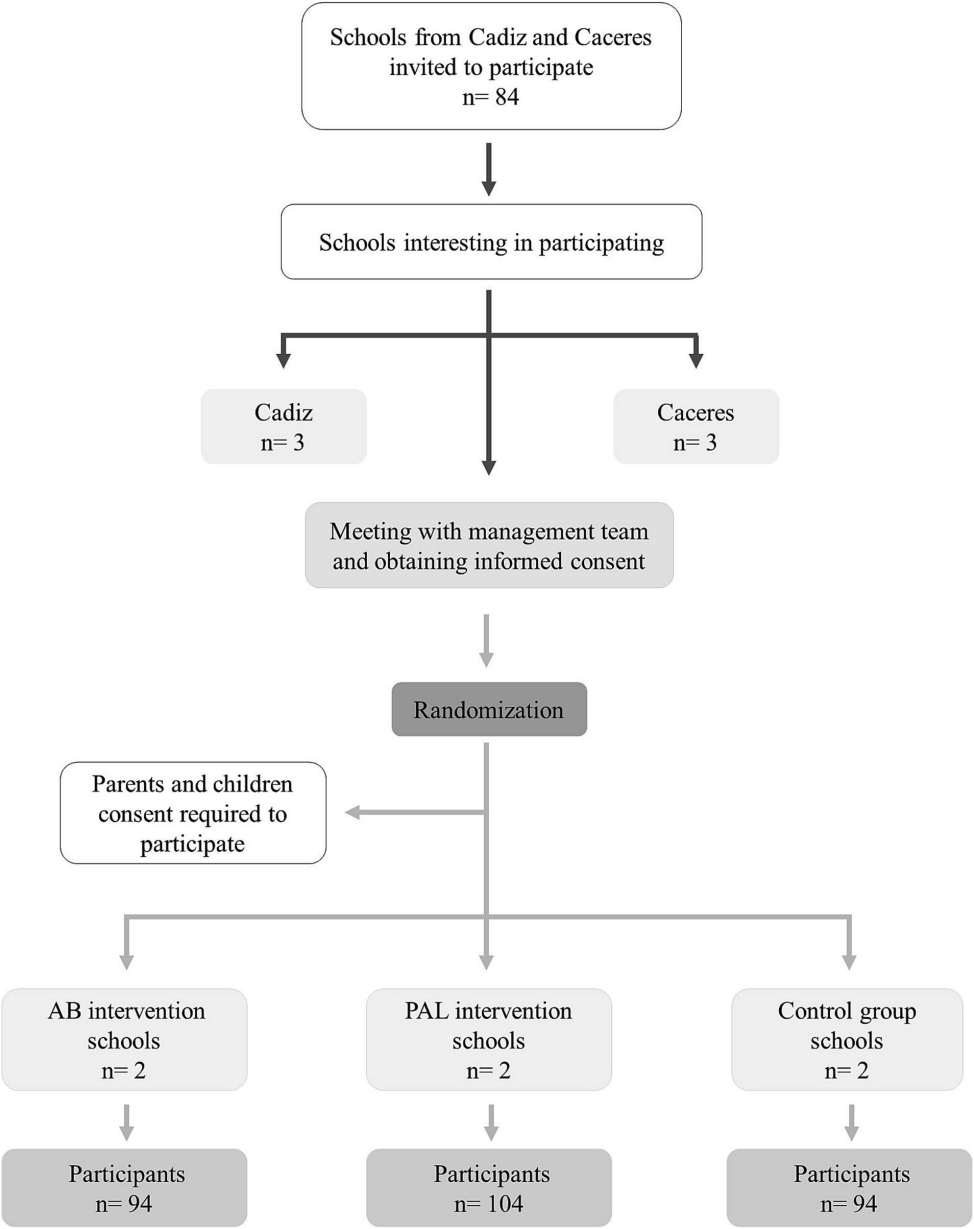
Follow diagram of study participants.

Figure 1 illustrates the flow diagram of the participants recruitment process. A total of 20 schools in Caceres and 64 schools in Cadiz are invited to participate through a letter of invitation addressed to the school’s management. A meeting is held with the management teams of the schools that accept the invitation, to explain the study and obtain their consent. From the total number of schools which accept to participate and provide their consent (three schools, both in Cadiz and Caceres), a randomization process at school level is conducted in each study province. Thus, three study groups are established in each city (Cadiz and Caceres): (i) AB intervention group, (ii) PAL intervention group, and (iii) control group. All students in the 7th or 8th grade of the participating schools who give their informed consent are invited to participate in the study. The parents of the students receive an information document describing the study, the inclusion criteria, the informed consent process, and an invitation to attend an information meeting at the school. To participate in the study, the parents or guardians must sign the informed consent form, along with the student’s consent.

A total of 292 participants were included in the study, ensuring effect sizes of 0.1 with an alpha level of 0.05 and a power of 0.80, even with an experimental dropout rate of 25% (GPower 3.1.9.4, Düsseldorf, Germany).

### 2.3 Interventions

The ACTIVE CLASS study implements two intervention programs over a period of 16-weeks: (i) AB, and (ii) PAL, consisting of including PA in academic lessons for secondary education, while a third group serves as a control.

#### 2.3.1 Active break intervention

AB intervention takes place daily and is carried out in the normal academic lessons, where two ABs per day are included in the 7th and 8th grade timetable of each secondary school. The timing of the AB is coordinated with the school management team. Whenever possible, one AB is scheduled before recess and the other after recess. It is important to note that no AB are scheduled during physical education classes or the lesson immediately following physical education. This ensures that the effect of the AB is not solely attributed to the PA performed in the preceding physical education class. The remaining class time follows the usual methodology without any variation.

The implementation of this intervention utilizes digital platform accessible to schools through individuals’ usernames and passwords. This platform has been specifically designed to facilitate the development of AB in school classes. An example of AB can be found in the following link: https://www.dropbox.com/s/dtye613hb3m3c50/VideoDescansoActivo.mp4?dl=0. This innovative tool allows to program different types of AB easily and quickly, by alternating variables such as physical exercise, its duration or intensity. Based on the findings from the review by Daly-Smith et al. (16), each AB has a duration of 5 min, of which 4 min of activity are used, including two sets of 20 s of work and 10 s of rest of four different exercises. Finally, a cool down consisting of deep breaths is performed. The activities selected for each of the AB include aerobic and strengthening activities (i.e., jumps, squats, lunges or skipping).

Teachers have access to the platform and are responsible for conducting the AB as scheduled by the research team. During the AB, students follow the instructions provided by an avatar that guides the session through the platform. To ensure the proper use of the platform, the research team provides training and support to the teachers. During the initial phase of the intervention, the research team accompanies the teachers for the first 2 weeks to ensure a smooth implementation of the AB and address any questions or concerns that may arise. This support aims to familiarize the teachers with the platform and ensure they feel confident in delivering the AB effectively.

#### 2.3.2 Physically active learning intervention

PAL intervention involves integrating PA into academic lessons, specifically within the subject of mathematics. This subject has a substantial body of evidence supporting its applicability for PAL, as it is a core subject taught in all grades of secondary education (26, 32, 33). Out of the total number of mathematics lessons taught per week in Spanish schools (four one-hour classes per week), one class per week for 16 weeks is dedicated to the PAL intervention. Each of the PAL is developed outside the classroom during the intervention.

The mathematics teacher corresponding to this intervention group, previously trained and with the constant support of the research team, oversees implementing the strategies in their academic lessons. Before each PAL class, the research team and the mathematics teacher collaborate in a meeting to determine the specific content to be covered and co-develop the activities to be included in the session. Some examples of activities used in the sessions can be found in the following free downloadable book: https://www.dykinson.com/libros/aprendizaje-fisicamente-activo-fundamentos-teoricos-y-estrategias-practicas-para-la-materia-de-matematicas-en-1o-y-2o-de-eso/9788411701617/

#### 2.3.3 Control group

The control group receives the usual academic lessons during the 16-weeks of intervention, without methodological modification or inclusion of breaks that could alter the usual levels of PA during school hours.

### 2.4 Measures

Participants are assessed at baseline (January 2023), post-intervention (May 2023), and 1 month after the intervention (June 2023) to test the stability over time of the strategies offered to teachers. Post-intervention measure of accelerometers-based variables and time-on-task are assessed during the last weeks of intervention, due to the characteristics of the indicators. To minimize variability in assessments, all measurements are conducted in schools by trained researchers who have received prior training.

#### 2.4.1 Primary outcome measures

##### 2.4.1.1 Physical activity and sedentary time

Actigraph (Actigraph GT3X+, Inc., Pensacola, FL, USA) is used to measure PA and sedentary time. Participants wear the monitor for eight consecutive days. To ensure a higher compliance rate when wearing the device, it was decided that participants would wear it on the non-dominant wrist, as this is an area that has been shown to be just as valid as on the waist (34). Participants are instructed to wear the accelerometer throughout the day (including sleeping hours), except for aquatic or situations where the device may come into contact with water. Sleep duration is captured through self-reported data, wherein each participant is provided with an accelerometer diary. Participants are required to diligently record the times at which they initiate sleep and awaken each day while wearing the accelerometer.

The screening and data collection procedures are conducted following the established protocols used in previous studies involving adolescents (34). Data will be downloaded and analyzed by using the ACTILIFE software (v.6.11.7 Actigraph TM). Inclusion criteria for the analyses are: (i) at least 3 days of valid weekday data and at least 1 day of valid weekend data; (ii) a minimum of 10 h of recording per day (34, 35).

#### 2.4.2 Secondary outcome measures

##### 2.4.2.1 Health-related physical fitness

Those tests comprising the High Priority ALPHA Health-Related Fitness Test Battery (36) are used to assess HRPF:

*20-m shuttle run test* is used to assess cardiorespiratory fitness. Two lines are marked on the track 20-m apart. Participants run back and forth between the lines, following a straight trajectory, and matching the rhythm of an audio recording. The initial speed is 8.5 km/h and is increased by 0.5 km/h per leg (each leg corresponds to 1 min). The test ends when the participant stops due to fatigue or when he does not cross the lines marked to the pace of the acoustic signals on two consecutive occasions. The test is performed once, and the last stage or half stage completed by the participant is scored.

*Hand grip test* is used to assess upper body maximal isometric muscular fitness. A validated hand-held dynamometer with an adjustable grip (TKK 5101 Grip D; Takey, Tokyo, Japan) is used. Prior to the test, the dynamometer’s grip is adjusted to the size of the participant’s hand (37). During the test, the participants stand upright and hold the dynamometer in one hand. They progressively squeeze the dynamometer until maximum force is developed, maintaining the pressure for at least 2 s, while ensuring that the elbow, arm, and trunk remain stable and unmoving. The test is performed twice, alternating between each hand. The maximum score achieved with each hand is recorded in kilograms, and the mean value between the two scores is calculated and saved for further analysis.

*Standing broad jump test* is used to lower body explosive muscular fitness. The participants stand behind a line with feet shoulder width apart. Then, with a slight swing, the participant is asked to jump forward as far as possible with both feet. If the participants rest his hands or lifts his feet off the ground on landing, the test is invalid. The test is performed twice, and the best score attained is recorded in centimeters for analyses.

*Body mass index and waist circumference* is used to assess body composition. For body mass measurement, participants stand barefoot on an electronic scale (type SECA 861; range, 0.05–130 kg; accuracy, 0.05 kg). Height measurement is taken in the Frankfort plane using a telescopic height measuring instrument (type SECA 225; range, 60–200 cm; accuracy, 1 mm). Each measurement is taken twice, and the mean of the two measurements is recorded. Body mass index is calculated as weight/height squared (kg/m2). On the other hand, waist circumference is measured using a non-elastic tape (SECA 200; range, 0–150 cm; accuracy, 1 mm). The tape is placed in the frontal plane at the midpoint between the superior iliac spine and the costal border at the mid-axillary line. The measurement is taken twice, and the mean of the two measurements is recorded.

Additionally, self-reported physical fitness level is measured through the International Fitness Scale (IFIS) (38). This scale has proven to be a valid instrument to correctly classify a sample of adolescents from different European countries according to their physical fitness levels (38). The scale is based on responses to five physical fitness questions: overall FP, cardiorespiratory fitness, muscle fitness, speed-agility, and flexibility. Participants are asked to rate each of the FP components on a 5-point Likert-type scale ranging from: “very poor” (1); “poor” (2); “acceptable” (3); “good” (4) and “very good.”

##### 2.4.2.2 Academic indicators

###### 2.4.2.2.1 School engagement

The engagement scale validated in adolescents and university students (UWES-S-9) (39, 40) is used to evaluate school engagement. The UWES-S-9 is composed of nine items that reflect the three dimensions of engagement: (i) vigor; (ii) absorption; and (iii) dedication, each dimension is represented by three items that are evaluated through a Likert-type scale, ranging from “never” (0 points) to “always” (6 points).

###### 2.4.2.2.2 Learning perception

Learning perception in mathematics is assessed by the questionnaire developed by Abella et al. (41). The questionnaire consists of eight items that measure two dimensions: “perceived learning” (items 1–4) and “satisfaction with learning” (items 5–8). Participants rate their agreement with each item on a five-point Likert scale, with “1” indicating “strongly disagree” and “5” indicating “strongly agree.”

###### 2.4.2.2.3 Academic performance

It is evaluated through the marks reported by the schools in the three official evaluations of the academic year, specifically in January, April, and June.

###### 2.4.2.2.4 Mathematical fluency test

Mathematical fluency test is assessed by means of test number 6 of the *Batería* III Woodcock-Muñoz™ (42) (age range 2–90 years old). This battery has been validated to specifically measure academic achievement in math. In this test, participants are given a three-minute period to perform as many simple mathematical calculations as possible.

###### 2.4.2.2.5 Time-on task

Time-on task is evaluated in mathematics classes or the time at which the AB are to be held and in the consecutive class 1 h later. Students’ on-task behavior was graded attending to the guidelines used Mahar et al. (43) in on-task and off-task. On one hand, on-task behavior is characterized by students making eye contact with the teacher and actively following the teacher’s instructions or class rules. On the other hand, off-task behavior is identified when students fail to pay attention or break class rules. In turn, off-task behavior is further categorized into three subtypes: (i) off-task-motor (any motor response that breaks the rules and/or disrupts the learning situation); (ii) off-task-noise (any oral response that breaks the classroom rules and/or disrupts the learning situation) and (iii) off-task-passive (moments when the student does not participate in any interaction or do anything when expected to participate).

During each assessment, a researcher observes six students. Each investigator has 15 s to observe and identify the type of behavior exhibited by each student, recording the observations on a worksheet before moving on to the next student. The investigators are in a spot where they can see the whole classroom properly. They use headphones and a prerecorded track with audible signals that indicate when to evaluate to the next student. Before data collection, the evaluators underwent a familiarization process with the measurement of this variable in the same context (i.e., teachers, students and classroom) to be assessed. This helped mitigate any potential reactivity effects that students and teachers might exhibit.

##### 2.4.2.3 Cognition

The executive functions of inhibition, cognitive flexibility and working memory are evaluated as main indicators of cognitive function. These are measured using the NIH Examiner program (44) through the (i) Flanker task; (ii) Shifting task and (iii) N-Back protocols, respectively.

###### 2.4.2.3.1 Flanker task

It evaluates the response inhibition and cognitive control (45). In this test, participants direct their attention to a fish located in the center of a row of five fish displayed on the screen. The fish in the center has a mark either above or below it. Each trial has a stimulus presentation time of 1,000 milliseconds. Participants are instructed to quickly indicate the direction in which the fish in the center is pointing.

###### 2.4.2.3.2 Shifting task

It evaluates cognitive flexibility (44). In this test, three figures are displayed on the screen, with one figure positioned at the top and one figure in each corner. The figures are presented in different colors. During the test, the word “SHAPE” or the word “COLOR” appears on the screen, accompanied by the computer reading it aloud. The participants have to associate the figure at the top with one of the figures in the corners. In case of hearing the word “COLOR” the participant should select the figure in the corner that has the same color as the top figure. On the contrary, in case of hearing the word “SHAPE,” the participants have to select the corner figure that has the same shape of the upper figure.

###### 2.4.2.3.3 N-back

It evaluates working memory (46). In this test, a first screen shows a white square located in a certain place, followed by a number that the participants are asked to read aloud. Subsequently, a second screen is shown with a white square that may be in the same or in a different location of the previous square. The participants should remember the location of the previous square.

##### 2.4.2.4 Psychological health

Health-related quality of life is measured through the EuroQol five dimensions three levels (EQ-5D-3L), which has been shown to be a viable, valid and reliable tool in children and adolescents (47). The EQ-5D-3L questionnaire measures health status across five dimensions: (i) mobility; (ii) self-care; (iii) usual activities; (iv) pain/discomfort and (v) anxiety/depression. Participants are asked to rate their level of difficulty or problems in each dimension using a Likert-type scale with three response options: (i) no problem, (ii) some problems, and (iii) many problems. This questionnaire also includes a visual analog scale (VAS) to assess general health, where the participants assign a score between 0 and 100 on the VAS to indicate their current perception of their overall health.

On the other hand, self-perceived health is measured through the classic self-reported health item (48), where participants have to classify their health among the following options: “excellent” (5); “very good” (4); “good” (3); “fair” (2) and “poor” (1).

##### 2.4.2.5 Motivational variables

*The Novelty Need Satisfaction Scale* (*NNSS*) was used to evaluate students novelty satisfaction (49). This scale has shown construct, discriminant and convergent validity along with measures of psychological need satisfaction and forms of motivation from self-determination. Participants are asked to rate their agreement with these five statements on a Likert-type scale ranging from 1 (strongly disagree) to 5 (strongly agree).

Enjoyment and boredom were evaluated with the *Spanish version of The Sport Satisfaction instrument (SSI)* (50). Although this scale was initially created for the sport contexts, the Spanish version was adapted to the physical education context (51), and its showed valid and reliable with adolescents. For this particular study, we replace the initial statement from physical education to math or general school context depending of the randomized group. The scale consists of eight items measuring intrinsic satisfaction, with two subscales: satisfaction/enjoyment (five items) and boredom (three items). Participants rate their agreement with the items related to fun or boredom on a five-point Likert-type scale, ranging from 1 (strongly disagree) to 5 (strongly agree).

##### 2.4.2.6 Teachers’ and students’ perception about the suitability and the development of physically active classes and active breaks

To gather information about the development of the intervention and gain insights into perceptions, barriers, areas for improvement, strengths, training needs, and sustainability, individual interviews and focus groups are conducted with teachers and students (52). Both individual interviews and focus groups are led by the same member of the research team in each study province.

Teachers involved in the PAL sessions (three teachers in Cadiz and three teachers in Caceres), as well as an equivalent sample of teachers in AB group, participate in one-hour semi-structured individual interviews. These interviews provide an opportunity for teachers to share their experiences, thoughts, and suggestions regarding the PAL and AB. In addition, a one-hour focus group is conducted with a sub-sample of six students from each class in both the AB and PAL groups. These focus groups allow students to express their perceptions, experiences, and opinions about the intervention, providing valuable insights into their engagement, enjoyment, and perceived benefits of the PAL and AB.

#### 2.4.3 Confounding variables

##### 2.4.3.1 Dietary patterns

Adherence to the Mediterranean diet is assessed using the updated version 2.0, previously validated in children and adolescents of the of the KIDMED questionnaire (53). The KIDMED 2.0. questionnaire consists of 16 questions that participants answer. The questions in the questionnaire have both positive and negative connotations in relation to the Mediterranean diet. Participants receive a score based on the sum of their responses, and the scores are classified into three levels: (i) ≥8, optimal Mediterranean diet; (ii) =4–7, improvement needed to adjust intake to Mediterranean patterns; (iii) ≤3, very low diet quality.

##### 2.4.3.2 Sociodemographic characteristics

Sociodemographic characteristics, specifically the socioeconomic status of the participants, are collected using the Spanish-adapted version III of The Family Affluence Scale (FAS III) (54), which has been presented as a valid instrument to measure the socioeconomic status of adolescent (54). This scale consists of six questions that participants have to answer related to the purchasing power of the participant’s family. Each question is scored on a categorical scale, and the sum of the scores from the six items result in an aggregate index ranging from 0 to 13. An overall view of the different measures at different time points is depicted in Supplementary material.

### 2.5 Data analysis

Data will be presented as mean and standard deviation or median and interquartile range, if applicable, for continuous variables, and frequency and percentage for categorical variables. As a cross-sectional fashion, descriptive, correlational, regression and differential analyses will be implemented. Before to implement the main analysis to test the effects of the interventions, we will test for group equivalence at baseline, by using independent samples *t*-test for continuous variables and chi square test for categorical indicators. To assess the effects of the two interventions on outcomes, repeated measured analysis will be used with the outcome measures as dependent variables in separate models, the intervention as an independent variable and controlling for potential confounders (i.e., gender). If normality analyses show non-normal distributions, the equivalent non-parametric analyses will be performed. Quantitative analyses will be completed using the SPSS 26 statistical package (IBM, Armonk, NY, USA), establishing a confidence level of 95% (*p* < 0.05).

On the other hand, the qualitative information from the semi-structured interview and the focus group will be transcribed and analyzed with the NVIVO software (55). These data will be analyzed following content analysis strategies where two phases will be differentiated. A first deductive phase aimed at detecting any type of information related to the purpose of the study. Subsequently, an inductive phase where the experiences, thoughts and reflections of teachers and students related to the AB and PAL will be identified (56).

All the analyses will be carried out considering intention to treat, keeping the participants in the group they were originally allocated and taking into consideration the CONSORT guidelines for cluster RCTs (57).

## 3 Discussion

This paper describes the protocol for a RCT that aims to test the effects of an intervention program based on the inclusion of PA, through AB and PAL, in academic classes on PA levels, sedentary time, HRPF, academic indicators, cognition, and psychological health markers in secondary education students.

In relation to AB and PAL methodologies, the limited studies conducted in secondary education fail to yield conclusive results. Specifically, in the case of AB, investigations within the secondary education context primarily center on the variables of time-on-task and cognition. However, disparate findings emerge concerning the impact of AB on time-on-task (18, 19), and the enhancement of cognitive variables (20). The discrepancies in the obtained results may be attributed to methodological nuances. Although the duration of AB (ranging from 4 to 10 min) was consistent across studies, variations in the duration of interventions [ranging from one (19), five (18), and eight (20) weeks] and frequency of application [ranging from two (18) to four (20) AB per day] were observed.

As for PAL, PA and academic performance, these take precedence as study outcomes in secondary education. Studies indicate an association of PAL with an improvement in school PA levels (24), but present mixed results concerning the effect of PAL on academic performance (25, 26). Methodological variations appear to underlie these discrepancies, with studies showing a positive PAL association tending to employ longer intervention periods, spanning from 2 to 7 months (24, 26). Moreover, as emphasized by Norris et al. (21), inconsistencies in reporting PAL interventions doses, including duration and frequency, further complicates result comparisons.

Given the prevalent issues of physical inactivity and sedentary lifestyles among school-aged youth (6–8), there is a pressing need for interventions such as the ACTIVE CLASS study to address this gap and explore the potential impact of AB and PAL. Anticipated results align with those observed in primary education, where the implementation of AB and PAL is positively associated with physical and psychological health (22, 23, 58–60), PA levels (16, 17, 21), time-on-task (16, 17, 21), and academic performance (21). Although significant changes in cognitive markers in children were not consistently observed in the scientific literature (16, 21, 61), it remains a variable with a potential improvement, given the positive association observed between PA levels and cognitive performance in children and adolescents (28).

### 3.1 Strengths and limitations

The present study has diverse strengths that should be mentioned: (i)the study is carried out on a sample of secondary education students. This is an educational stage which has received less attention in the scientific literature than primary and preschool education; (ii) the PAL intervention involves a collaborative approach between mathematics teachers and researchers, ensuring a higher level of sustainability of the intervention. This co-development process enhances the integration of PA into academic lessons and promotes long-term adoption by teachers; (iii) the study design will bring new evidence allowing to control the internal validity of the results. In addition, to corroborate the stability over time of the strategies offered, a follow-up measure of 4 weeks is incorporated at the end of the intervention.

On the other hand, some limitations should be mentioned: (i) the generalizability of the study results may be limited to the specific school context and characteristics of the participants included in the study (i.e., facilities, teachers, student motivation, etc.); (ii) the stability of the interventions may be compromised once the research team’s support is no longer available. Maintaining the AB and PAL interventions in real school settings without the continued support of the research team can be challenging.

## 4 Conclusion

The ACTIVE CLASS study examines the effectiveness of including PA in secondary education academic lessons through AB and PAL on PA level and sedentary time, HRPF, academic indicators, cognition, psychological health, and motivational variables. The implementation of this RCT has the potential to provide useful and innovative training to the educational community for the implementation of educational methodologies and strategies that facilitate higher academic performance and a better quality of life for their students.

## Ethics statement

The studies involving humans were approved by Bioethics Committees of the Andalusian Government (Cadiz, Spain), and the Bioethics and Biosafety Committees of the University of Extremadura (UEX) (Caceres, Spain). The studies were conducted in accordance with the local legislation and institutional requirements. Written informed consent for participation in this study was provided by the participants’ legal guardians/next of kin.

## Author contributions

MG-P: Writing – review & editing. DS-O: Writing – review & editing. AG-C: Writing – review & editing. EC-C: Writing – review & editing. FM-A: Writing – review & editing. RM-G: Writing – review & editing. FB-C: Writing – review & editing. AR-H: Writing – review & editing. MV-S: Writing – review & editing. CP-M: Writing – review & editing. JC-C: Writing – review & editing. VS-J: Writing – review & editing. IG-P: Writing – review & editing. TG-C: Writing – review & editing. JC-P: Writing – review & editing. DC-M: Writing – review & editing.
