# Supplementary material for: Effects of the inclusion of physical activity in secondary education academic classes on educational indicators and health markers: rationale and methods of the ACTIVE CLASS study
Source: Front Public Health. 2024 Jan 5;11:1329245. doi: 10.3389/fpubh.2023.1329245 (PMC10796663; doi:10.3389/fpubh.2023.1329245)
Supplement: Supplementary file 1 [file Table_1.DOCX]

| Measure | Pre-intervention  (January-February 2023) | | |  | Intervention  (February-May 2023) | | |  | Post-intervention  (May 2023) | | |  | Follow-up  (June 2023) | | |
| --- | --- | --- | --- | --- | --- | --- | --- | --- | --- | --- | --- | --- | --- | --- | --- |
| Intervention group | AB | PAL | Control group |  | AB | PAL | Control group |  | AB | PAL | Control group |  | AB | PAL | Control group |
| Variables: |  |  |  |  |  |  |  |  |  |  |  |  |  |  |  |
| Health indicators: |  |  |  |  |  |  |  |  |  |  |  |  |  |  |  |
| PA and sedentary time | X | X | X |  | X | X | X |  |  |  |  |  |  |  |  |
| Health-related fitness | X | X | X |  |  |  |  |  | X | X | X |  | X | X | X |
| Academic indicators: |  |  |  |  |  | | |  |  |  |  |  |  |  |  |
| *School engagement* | X | X | X |  |  |  |  |  | X | X | X |  | X | X | X |
| *Learning perception* | X | X | X |  |  |  |  |  | X | X | X |  | X | X | X |
| *Academic performance* | X | X | X |  |  |  |  |  | X | X | X |  | X | X | X |
| *Mathematical fluency* | X | X | X |  |  |  |  |  | X | X | X |  | X | X | X |
| *Time-on-task* | X | X | X |  | X | X | X |  |  |  |  |  |  |  |  |
| Cognition: |  |  |  |  |  | | |  |  |  |  |  |  |  |  |
| *Inhibition* | X | X | X |  |  |  |  |  | X | X | X |  | X | X | X |
| *Cognitive flexibility* | X | X | X |  |  |  |  |  | X | X | X |  | X | X | X |
| *Working memory* | X | X | X |  |  |  |  |  | X | X | X |  | X | X | X |
| Psychological health: |  |  |  |  |  | | |  |  |  |  |  |  |  |  |
| *Health status* | X | X | X |  |  |  |  |  | X | X | X |  | X | X | X |
| *Self-perceived health* | X | X | X |  |  |  |  |  | X | X | X |  | X | X | X |
| Motivational variables: |  |  |  |  |  | | |  |  |  |  |  |  |  |  |
| *Novelty* | X | X | X |  |  |  |  |  | X | X | X |  | X | X | X |
| *Enjoyment and boredom* | X | X | X |  |  |  |  |  | X | X | X |  | X | X | X |
| Dietary patterns | X | X | X |  |  |  |  |  | X | X | X |  | X | X | X |
| Sociodemographic characteristics | X | X | X |  |  |  |  |  | X | X | X |  | X | X | X |
| Qualitative information |  |  |  |  |  |  |  |  | X | X | X |  |  |  |  |
